# The *Porphyromonas gingivalis* lipid A 1-phosphatase LpxE requires a functional type IX secretion system for its activity

**DOI:** 10.1080/20002297.2025.2600179

**Published:** 2025-12-14

**Authors:** Sunjun Wang, Yichao Liu, Beichang Zhang, Joseph Aduse-Opoku, Roberto Buccafusca, Oscar Ayrton, Giulia Mastroianni, Pedro Machado, Mark A. J. Roberts, Michael A. Curtis, James A. Garnett

**Affiliations:** a Centre for Host-Microbiome Interactions, Faculty of Dentistry, Oral & Craniofacial Sciences, King’s College London, London, UK; b School of Physical and Chemical Sciences, Queen Mary University of London, London, UK; c Department of Chemistry, Faculty of Natural, Mathematical & Engineering Sciences, King’s College London, London, UK; d Centre for Ultrastructural Imaging, King's College London, London, UK; e Department of Biochemistry, University of Oxford, Oxford, UK

**Keywords:** *Porphyromonas gingivalis*, type IX secretion system, outer membrane vesicle, lipopolysaccharide, lipid A

## Abstract

**Background:** *Porphyromonas gingivalis* is a Gram-negative bacterium that plays a central role in the development of periodontal disease. It uses a type IX secretion system (T9SS) to export virulence factors to the bacterial surface where they are attached to A-LPS, one of the two forms of lipopolysaccharide (LPS) produced in *P. gingivalis*, and then packaged into outer membrane vesicles (OMVs). We previously showed that 1-P dephosphorylation of the lipid A component of LPS is regulated by the T9SS outer membrane protein PorV, and this is linked to membrane destabilisation and OMV blebbing/formation.

**Objective:** This study aimed to investigate whether other T9SS outer membrane proteins are required for correct OMV biogenesis.

**Design:** We examined gingipain activity, gingipain secretion, A-LPS production, OMV morphology, and lipid A structure in *P. gingivalis* W50, T9SS mutant strains, and a lipid A 1-phosphatase (Δ*lpxE*) mutant strain.

**Results:** A functional T9SS is required for LpxE activity and correct vesicle formation, and this is likely through the function of an exported type IX-cargo protein.

**Conclusion:** This study provides insight into a new mechanism that links type IX cargo sorting with OMV blebbing, which may also be present in other *Bacteroidota* that colonise the gut and oral cavity.

## Introduction

Periodontitis is a biofilm-associated inflammatory disease which affects the supporting tissues around the teeth and is linked with a range of chronic diseases including cardiovascular disease, diabetes, rheumatoid arthritis, and pregnancy complications [1,2]. The global incidence of severe periodontitis is estimated at 11% and it is ranked as the sixth most prevalent disease worldwide. *Porphyromonas gingivalis* is a major microbial agent of periodontitis [3], and it uses a type IX secretion system (T9SS) to export key virulence factors onto the bacterial surface [4,5]. Subsequently these can be sorted into outer membrane vesicles (OMVs) [6], which are spherical structures that bleb from the outer membrane and can transport virulent traits long distances from their source [7]. For example, the lysine-specific (Kgp) and arginine-specific (Rgp: RgpA and RgpB) gingipain cysteine proteases are exported to the *P. gingivalis* surface and OMVs [8], where they carry out a range of functions that include host adhesion, modulation of host immunity, and degradation of host proteins and tissues [9].

The T9SS is produced by a wide range of bacteria across the *Fibrobacteres-Chlorobi-Bacteroidetes* superphylum and is highly versatile. While in *P. gingivalis* its main function is the export of virulence factors, in *Flavobacterium johnsoniae* it enables gliding motility [10], and in *Tannerella forsythia* it supports S-layer formation [11]. The T9SS is composed of two major structures: a >1.4-MDa ‘translocon complex’, which crosses both the inner and outer membranes [12,13], and a smaller ‘attachment complex’, located in the outer membrane [14]. Within the translocon complex, PorL forms a pentamer in the inner membrane which can generate proton motive force and rotate a dimer of PorM, which extends across the periplasm [15–17]. A dodecamer of PorL/PorM sub-complexes are arranged as a tube-like structure, with PorM binding PorN, which forms an oligomeric ring with PorK on the periplasmic face of the outer membrane [13,18]. Above the PorK/PorN ring on the extracellular surface there are several copies of the tranlocon pore protein, Sov [13]. These associate with a *β*-barrel outer membrane protein (OMP), PorV, a periplasmic lipoprotein, PorW, and a periplasmic protein, SkpA, which anchor Sov to the PorK/PorN ring, and binds a protein of unknown function, PorD, respectively [13,19–21].

All T9SS cargo proteins contain a conserved Ig-like C-terminal domain (CTD) [22–25], and after entering the periplasm via the SEC pathway, the CTD is directed into the Sov channel through interactions with PorM, PorN, SkpA, PorW and PorV [20,26]. Although the precise mechanism is unclear, it has been proposed that transfer of proton motive force from the inner membrane to the PorK/N ring may stimulate the lateral release of cargo proteins from Sov [20]. This results in the association of a periplasmic plug protein inside Sov to prevent leakage from the periplasm [21] and the release of a binary PorV/cargo complex in the outer membrane [25], which acts as a shuttle to move cargo proteins away from Sov to an attachment complex [14]. The attachment complex is formed from PorQ, PorU, PorV, and PorZ in a 1:1:1:1 stoichiometric ratio. PorQ is another OMP, and PorQ and PorV tether PorZ and PorU, respectively, to the bacterial surface [12,14,27].

Lipopolysaccharide (LPS) is the major component of the outer membrane of Gram-negative bacteria and it is composed of lipid A, a core oligosaccharide, and a distal O-antigen of repetitive glycan polymers [28]. *P. gingivalis* produces two forms of LPS, conventional neutral O-antigen polysaccharide-linked LPS (O-LPS) and anionic-LPS (A-LPS) which has an anionic O-antigen polysaccharide [29]. Within the attachment complex, PorZ has a role in presenting A-LPS to PorU, a sortase, which then cleaves off the cargo CTD and mediates A-LPS attachment to the C-terminus of the truncated cargo via a short carbohydrate linker [30–34].

In addition, other OMPs have also been reported to be essential for correct type IX secretion. PorP is an OMP that interacts with the PorK/PorN ring and through association with the periplasmic lipoprotein PorE, it is thought to anchor the T9SS to peptidoglycan [35,36]. PorP also binds type B CTDs/cargo and attaches them to the bacterial surface in an A-LPS-independent manner [35]. Likewise, PorG, PorT and PorF are also OMPs that are essential for correct functioning of the T9SS, but while PorG has been shown to interact with the PorK/PorN ring [18,37], the interaction profiles of PorT and PorF are unknown [22].

Many bacteria can express lipid A phosphatase and/or *O*-deacylase enzymes in their inner and outer membranes, respectively, which promote bacterial evasion of host immunity but also alters the properties of their outer membrane [28]. In *P. gingivalis*, one lipid A 1-phosphatase (LpxE/PG1773), two lipid A 4′-phosphatases (LpxF1/PG1587, LpxF2/PG1738), and one 3′-*O*-deacylase (LpxR/PG1333) have been identified. These have been implicated in *P. gingivalis* evasion of Toll-like receptor 4 (TLR4) sensing through the production of dephosphorylated/deacylated forms of lipid A [38–41]. However, we have also shown that deletion of *porV* in *P. gingivalis* W50 strain results in abrogation of lipid A 1-phosphatase activity, and deformation of OMVs [42]. In this study, we aimed to understand whether the regulation of LpxE activity was specific to PorV function or whether this regulation was a more general consequence of type-IX secretion. We demonstrate that disruption of the attachment complex and other T9SS OMPs in *P. gingivalis* W50 also cause major defects in T9SS function and LpxE activity and produce larger/deformed OMVs. In addition, an Δ*lpxE* strain generates larger/deformed OMVs, displays defects in protein accumulation in both the outer membrane and OMVs, and we show that *P. gingivalis* LpxE is predicted to be a unique outer membrane lipid A 1-phosphatase that contains a distinct ~200 amino acid extension at its C-terminus. We have identified similar LpxE enzymes in other *Bacteroides* species that colonise the gut and oral cavity and we propose that in *P. gingivalis* a cargo protein exported by the T9SS is able to regulate the activity of LpxE, and destabilisation of the outer membrane through lipid A modification/A-LPS repulsion is important for OMV development.

## Materials and methods

### Bacterial strains and media

All strains used in this study are listed in **Supplementary Table 1**. *P. gingivalis* W50 strain served as wild type and parent for all mutants and were routinely grown on blood agar plates containing 5% (v/v) defibrinated horse blood or in brain heart infusion (BHI) broth supplemented with hemin (5 μg/ml), in an anaerobic atmosphere of 80% N_2_, 10% H_2_, and 10% CO_2_ (Don Whitely Scientific). Clindamycin (5 μg/ml) or tetracycline (1 μg/ml) was added when required. *Escherichia coli* NEB 5-alpha (New England Biolabs) was used for plasmid maintenance and cloning.

### Gene deletions

Single isogenic mutants defective in *porU*, *porQ*, *porZ*, *porP*, *porT*, *porG, porF* and *lpxE* were generated using primer pairs designed to separately amplify the 5′ and 3′ ends of each ORF by PCR (**Supplementary Table 2**). Following purification and digestion with SacI and XbaI, amplicons were ligated to an *erm* cassette, retrieved from plasmid pVA2198 [43] by T4-DNA ligase. The mixture was purified and used as a template in PCR to generate linear chimeric amplicons that comprise *erm* cassette flanked by the 5′ and 3′ regions of the ORF, which were then electroporated into exponential cells of *P. gingivalis* W50 to generate clindamycin resistant mutants by allelic exchange [44,45]. *P. gingivalis* mutant colonies were then screened by PCR to identify erm cassettes that had been inserted in the correct position. For complementation of *lpxE*, an amplicon corresponding to the *lpxE* ORF and an additional 500 bp regulatory upstream sequence was amplified from W50 genome by PCR and cloned into the pUCET1 complementation plasmid [46] using BglII and NotI sites. The plasmid was linearised with XbaI and the flanking *erm* cassette was then used to target the homologous regions in *lpxE* mutant via electroporation and allelic exchange with selection for the tagged *tetQ* on blood agar plates [29].

### Pigmentation assay

*P. gingivalis* W50 and mutant strains were first grown anaerobically for 24 h in BHI broth supplemented with hemin (5 μg/ml) at 37 °C. These were then streaked onto blood agar plates and incubated anaerobically at 37 °C for 7 days.

### Gingipain activity assay

*P. gingivalis* W50 and mutant strains were grown in BHI broth supplemented with hemin (5 μg/ml) in an anaerobic cabinet for 24 h. Whole cell cultures were either assayed straight away or were centrifuged at 9,000 × g, 4 °C for 25 min and the supernatant retained. Arg- and Lys-specific protease activities were then measured at 30 °C in a 1.0 ml reaction volume containing 0.1 M Tris-HCl pH 8.0, 10 mM L-cysteine, 10 mM CaCl_2_, with either 0.5  mM Nα-benzoyl-DL-Arg-*p*-nitroanilide (DL-BR*p*NA) or 0.5 mM *N*-*α*-acetyl-L-lysine-*p*-nitroanilide (L-AcLys*p*NA) as the substrate, respectively. The reaction was monitored at 405 nm, and enzyme activity was expressed as increase in absorbance/min/OD_600 nm_ in a cell culture at 30 °C.

### Bacterial fractionation

*P. gingivalis* W50 and Δ*lpxE* strains were grown in 20 ml of BHI broth supplemented with hemin (5 μg/ml) in an anaerobic cabinet for 48 h. Cells were then centrifuged at 17,000 × g, 4 °C for 15 min and the supernatant (OMV enriched sample) was applied to a 0.2 μm syringe filter to remove any remaining cells. Cell pellets were then washed twice in 5 ml of 50 mM Tris-HCl pH 8, 50 mM NaCl, 5 mM CaCl_2_ and centrifuged at 900 × g and 4 °C for 10 min, and after repeating, resuspended in 10 ml of PBS with 10 mM EDTA, and heated in a water bath at 60 °C for 30 min. After chilling on ice, the cell suspensions were sonicated with intermittent cooling on ice and then centrifuged at 17,000 × g at 4 °C for 10 min to remove unbroken cells and cell debris. The supernatant was then centrifuged at 48,400 × g at 4 °C for 60 min and the pellet was resuspended in 50 mM Tris-HCl pH 8, 50 mM NaCl, 5 mM CaCl_2_, 0.5% (w/v) sarcosine, with constant stirring at room temperature for 30 min. After centrifuging at 48,400 × g for 30  min, the outer membrane sample was prepared by reconstituting the pellet in 720 μl of 50 mM Tris-HCl pH 8, 50 mM NaCl, 5 mM CaCl_2_, 1% (v/v) Triton X-114.

### Immunoblotting

Samples were run on a NuPAGE 4–12% SDS-PAGE gel (Invitrogen), followed by transfer onto a PVDF membrane using the semi-dry Invitrogen iBlotter and iBlotter transfer blotting solution. The membrane was incubated in 3% (w/v) BSA in TBST at 4 °C overnight, washed three times with TBST and then incubated with primary antibody (rabbit Rb7, rabbit mAb CTD or mouse mAb 1B5) [25,44,47] diluted 1:1,000 in blocking buffer for 2 h at room temperature. The membrane was then washed three times with TBST for 10 min and incubated with the secondary antibody diluted to 1:2,000 at room temperature for 2 h. After three, 10 min washes with TBST buffer, membranes were treated with enhanced chemiluminescence substrate (ECL; Pierce) before detection by chemiluminescence (Bio-Rad, ChemDoc).

### Nanosight particle analysis

Five millilitres cultures of *P. gingivalis* W50 and mutant strains were grown in BHI broth overnight, adjusted to OD_600_ 2.0, and then centrifuged at 26,000 × g and 4 °C for 30 min. The supernatant was filtered with a 0.22-μm filtration apparatus. The filtrate containing OMVs was diluted 10-fold and subjected to Malvern NanoSight LM10 nanoparticle analysis using a laser light source with wavelengths of 405, 532 and 638 nm, and the particles were tracked and sized.

### Transmission electron microscopy

*P. gingivalis* W50 and derivatives were grown at 37 °C in BHI broth supplemented with hemin (5 μg/ml) in an anaerobic cabinet for 24 h. These were then fixed in 100 mM phosphate buffer pH 7.0 containing 3% (w/v) glutaraldehyde, 1% (w/v) formaldehyde and 0.5% (w/v) tannic acid, washed with 100 mM phosphate buffer pH 7.0, and then incubated overnight in 100 mM phosphate buffer pH 7.0 containing 2% (w/v) osmium tetroxide. Bacterial cells (10 μl) were then applied to mesh copper grids, prepared with glow discharged carbon support films, incubated for 2 min, washed five times with 50 μl of 1% aqueous uranyl acetate, and then left to dry for 5 min. Electron micrographs were taken using a JEOL 1230 transmission electron microscope operating at 80 kV. Alternatively, cultures were concentrated by centrifugation for 5 min at 2,000 × g, loaded in gold plated carriers and then frozen at high-pressure in a Leica EM ICE high-pressure freezer (Leica Microsystems). For ultrastructural analysis, the samples were freeze substituted in a solution of acetone containing 2% (v/v) osmium tetroxide, 0.1% (v/v) uranyl acetate, and 5% (v/v) distilled water in the Leica AFS. The samples remained at −90 °C for 10 hrs, and subsequently warmed to –20 °C over a period of 12  hrs. Samples were transferred to 4 °C for 30  min, followed by washing with anhydrous acetone at room temperature. The samples were infiltrated and embedded in Spurr resin. Sections of 70 nm were collected using a Leica UC7 ultramictrome, followed by post-staining with lead citrate. Electron micrographs were then taken using a JEOL JEM 1400 Flash instrument running at 80 kV. Images were then analysed to determine the mean cell and OMV diameters, where OMVs were within 50 nm of blebbing cells.

### Mass spectrometry

Bacteria were cultured for 48 h in BHI medium containing 5 μg/ml hemin. LPS was isolated using a modified version of the Tri-Reagent protocol for LPS isolation [48]. Specifically, bacterial cells were harvested at 12,000 × g for 30 min and resuspended in 2 ml PBS, washed twice in 2 ml H_2_O and then lyophilised. These were then resuspended in 20 ml of Tri-Reagent per mg of cells and incubated at room temperature for 15 min. A total of 20 ml of chloroform per mg of cells was added to create a phase separation and the mixture was vortexed and incubated at room temperature for a further 10 min. The resulting mixture was centrifuged at 12,000 × g for 10 min and the aqueous phase, containing LPS, was removed and retained. Then, 100 ml H_2_O was added to the remaining organic phase and the process was repeated twice. The aqueous phase samples were then combined and lyophilised.

To generate lipid A, dried LPS samples were resuspended in 10 mM sodium acetate pH 4.5 containing 1% (w/v) sodium dodecyl sulphate (SDS). The solution was heated at 100 °C for 1 h, followed by lyophilisation overnight. The resulting lipid A pellets were washed once in ice-cold 95% ethanol containing 0.02 *N* HCl and then three times in 95% ethanol, followed by a final extraction with 1,160 μl of chloroform-methanol-water (1:1:0.9 v/v/v) to remove residual carbohydrate contaminants. The chloroform layer containing the lipid A was dried and used for MALDI-TOF MS. Samples purified from W50 and T9SS mutants were dissolved in 10 μl of 20 mg/ml 5-chloro-2-mercaptobenzothiazole (CMBT) in chloroform-methanol at 1:1 (v/v) and 0.5 μl of each sample was analysed in negative-ion mode on an AutoFlex Analyser (Bruker Daltonics) [49]. Lipid A isolated from the Δ*lpxE* strains was dissolved in 10 μl of 10 mg/ml norharmane (9H-pyrido[3,4]indole) in methanol-water at 2:1 (v/v) and 0.5 μl of sample was analysed in negative-ion mode on a Shimadzu AXIMA iDplus Performance MALDI-TOF/TOF mass spectrometer.

### RT-qPCR

Wild type *P. gingivalis* W50 and derivatives were grown anaerobically in BHI medium containing 5 μg/ml hemin for 6  hrs at 37 °C, from a starter culture with initial OD_600 nm_ = 0.5. Cell pellets were resuspended in 100 μl of freshly prepared 10 mM Tris-HCl pH 8.5 containing 1 mg/ml lysozyme and incubated at room temperature for 5 min. Total RNA was then extracted using a Monarch Total RNA Miniprep Kit (NEB) and cDNA was synthesised via reverse transcription using an iScript cDNA Synthesis Kit. The reaction mixture was used directly as a cDNA template for RT–qPCR performed in a Rotor-Gene 6,000 real time cycler (Corbett Research) using HOT FIREPol EvaGreen qPCR Mix Plus (Solis BioDyne) and with the primers shown in **Supplementary Table 2**.

### Bioinformatics analysis

The primary sequence for *P. gingivalis* LpxE (Uniprot ID: B2RLI7) was analysed using the BLASTp server [50] using a threshold of 0.05, Matrix: BLOSUM62, Gap Costs: Existence 11 Extension 1, Compositional adjustments: Conditional compositional score matrix adjustment. Sequences from unique strains with an accession length >450 residues (**Supplementary Table 3**) were then compared using the M-Coffee function within the T-Coffee multiple sequence alignment server [51]. SignalPv6.0 was used for signal sequence predictions [52], PSIPREDv4 was used for secondary structure prediction [53], DeepTMHMM was used for transmembrane prediction [54] and DeepSoluE was used for solubility analysis [54]. For prediction of ΔG_app_ values for the transmembrane helices of LpxE PAP2 domains, AlphaFold models were downloaded from the AlphaFold protein structure database [55,56] and superposed onto the crystal structure of *Aquifex aeolicus* LpxE (PDB ID: 6ebu) [57]using PyMol [58]. The five transmembrane helices in *A. aeolicus* LpxE were defined using the Protein Data Bank of Transmembrane Proteins [59] and these were then mapped to the superposed AlphaFold models. Prediction of ΔG_app_ for each transmembrane sequence was then performed using the totaliser function in the Membrane Protein Explorer programme [60].

## Results

### T9SS OMP mutants are defective in gingipain secretion

**Figure 1.**
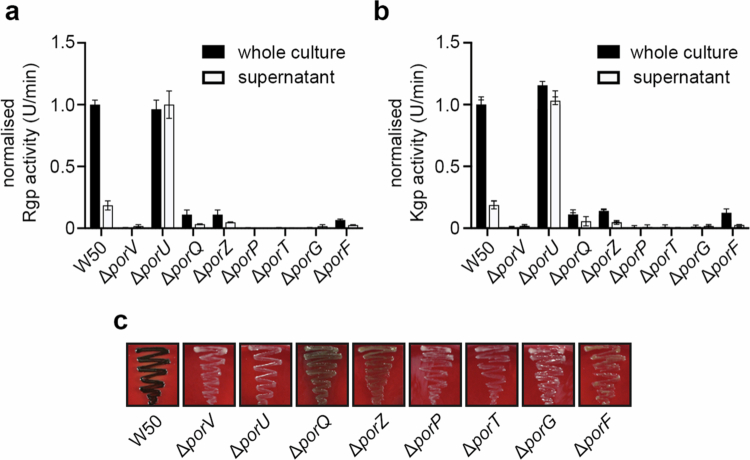
Gingipain activities of *P. gingivalis* T9SS OMP mutants. (a) R‐gingipain and (b) K‐gingipain activities in whole cultures and supernatants of *P. gingivalis* W50 and isogenic mutant strains, grown in BHI broth for 24 h. Rgp and Kgp activities were measured using substrates DL‐BR*p*NA and L‐AcK*p*NA, respectively, and are normalised to WT whole cultures. Data are presented as mean values +/− standard error of the mean (SEM) derived from *n* = 2 biologically independent experiments. (c) Pigmentation of *P. gingivalis* strains grown on blood agar plates for 7 days.

We previously constructed a Δ*porV* mutant in *P. gingivalis* W50, and we initiated this study by creating additional knockout mutants in the other known T9SS *β*-barrel OMPs (i.e., Δ*porQ*, Δ*porP*, Δ*porT*, Δ*porG*, and Δ*porF*) [27], but also Δ*porU* and Δ*porZ*, which are integral components of the outer membrane attachment complex [22]. To confirm that these mutant strains were defective in type-IX secretion we examined Arg- and Lys-gingipain activities in both whole cell cultures and isolated culture supernatants, the latter containing soluble gingipains and those enriched within OMVs (Figure 1a and b). For both Arg- and Lys-gingipain activities measured from wild-type W50, ~80% total activity was observed on the bacterial surface, while ~20% was observed in the supernatant. Conversely, no activity could be detected for the Δ*porV*, Δ*porP*, Δ*porT* and Δ*porG* strains, and there was an ~90% drop in the whole cell culture and supernatant activities for the Δ*porQ,* Δ*porZ* and Δ*porF* strains compared to W50. Although the Δ*porU* strain displayed whole cell culture activities comparable with wild type W50, this was primarily localised in the supernatant and indicated that this strain was impaired in retaining gingipains on the bacterial surface.

We next monitored gingipain export using a colony pigmentation assay. *P. gingivalis* displays black colony pigmentation when grown on blood agar due to Kgp gingipain activity and heme accumulation on the bacterial surface, and when these mutant strains are grown on blood agar, they all exhibited pigmentation defects (Figure 1c). While the Δ*porV*, Δ*porU*, Δ*porP*, Δ*porT* and Δ*porG* strains produced white colonies after 7 days growth, indicative of an inactive T9SS, the Δ*porQ*, Δ*porZ* and Δ*porF* strains instead formed beige colonies, reflecting an impaired export of Kgp due to reduced secretion and/or surface attachment. Analysis of the growth rates of these T9SS mutant strains in liquid media also demonstrated significant defects in the logarithmic, stationary and death phases of growth compared to W50, highlighting their important role in the normal growth cycle of *P. gingivalis* (**Supplementary Figure 1**)*.*

**Figure 2.**
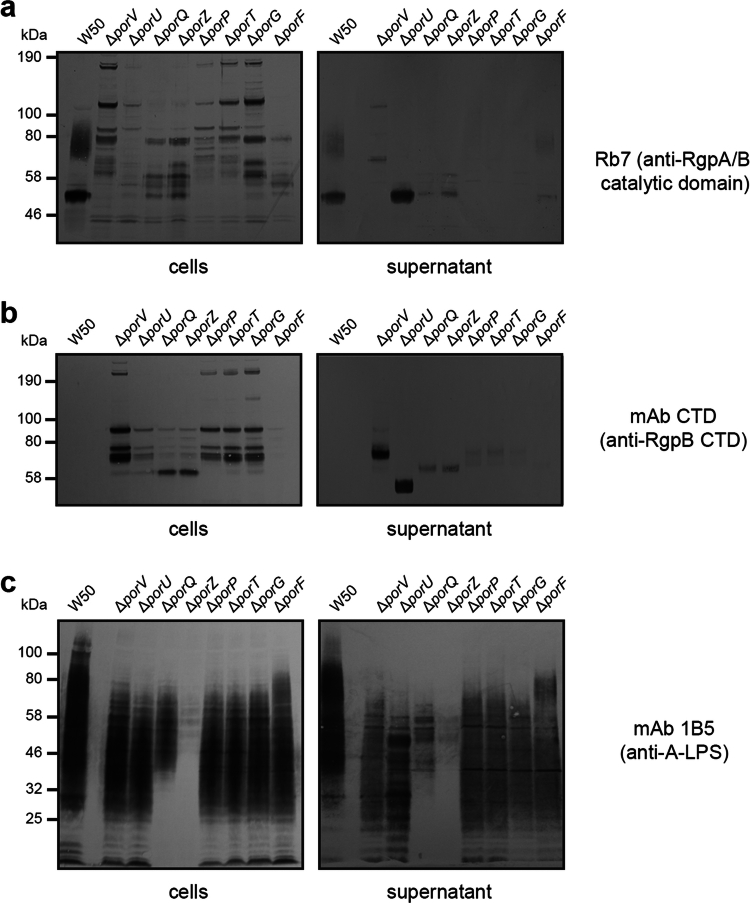
Immunoblots of *P. gingivalis* T9SS OMP mutants. Western blotting of *P. gingivalis* W50 and mutant strain cells and supernatants. (a) Antisera Rb7 against the RgpA/B catalytic domain, (b) mAb CTD against the RgpB CTD and (c) mAb 1B5 against A-LPS.

Strains were then separated into pelleted cells and culture supernatants and immunoblot analysis was carried out using antisera Rb7, which recognises the Rgp catalytic domain [44]. In both blots of WT W50, mature RgpB appeared as a diffuse band between ~70 and 90 kDa due to its attachment to A-LPS on the outer membrane and OMV surfaces (Figure 2a). We detected no mature gingipains in any of the mutant strains but instead observed higher molecular weight species which likely correspond to partially processed pro-proteins resulting from defective secretion. Moreover, in the whole cell samples, the Δ*porV*, Δ*porP*, Δ*porT* and Δ*porG* strains displayed similar banding, as did the Δ*porQ* and Δ*porZ* strains, while the Δ*porU* and Δ*porF* strain produced more unique profiles. In the supernatant, the Δ*porV* strain again displayed defective gingipain processing, and while RgpB was observed in the Δ*porU*, Δ*porQ* and Δ*porZ* mutants, it was not attached to A-LPS. No RgpA/B was detected in the supernatant from the Δ*porP*, Δ*porT* and Δ*porG* mutants, while mature RgpB was present in the Δ*porF* strain but at lower levels.

Immunoblotting was next carried out using monoclonal antibodies against the RgpB CTD (mAb CTD) and the anionic O-antigen of A-LPS (mAb 1B5) [25,47]. Analysis of W50 showed no detection of the free CTD in either whole cells or supernatants; likely due to proteolysis upon its release from cargo proteins during A-LPS attachment (Figure 2b). However, in all mutant strains except Δ*porF*, bands were detected at a higher molecular weight to the free CTD (~9 kDa), and represented partially processed pro-RgpB species, which lack A-LPS attachment and removal of the CTD. In both the whole cell and supernatant blots, while Δ*porU* appeared unique, Δ*porV*, Δ*porP*, Δ*porT* and Δ*porG* strains, and Δ*porQ* and Δ*porZ* strains, each produced similar band patterns, indicating that proforms of cargo proteins are retained in the cell in these mutants. Moreover, the detection of weak bands in the Δ*porQ*, Δ*porZ*, Δ*porP*, Δ*porT* and Δ*porG* mutants, and strong bands in the Δ*porV* and Δ*porU* mutants indicates that proforms of cargo proteins are released from the cell surface prior to A-LPS attachment or removal of the CTD. Of note, the single band detected in the Δ*porU* mutant supernatant is the expected molecular mass for mature RgpB prior A-LPS attachment. Finally, in the 1B5 blots of W50 cells and supernatants, A-LPS presented as a diffuse band ranging from ~25 to 150 kDa (Figure 2c), while in all mutants A-LPS was not detected above ~75 kDa (potentially A-LPS attached to cargo). However, in both cell and supernatant samples there was an additional decrease in A-LPS species below ~40 kDa in the Δ*porQ* strain, with an almost complete loss of A-LPS detection in the Δ*porZ* strain.

### *P. gingivalis* produces two distinct distributions of OMV sizes

**Figure 3.**
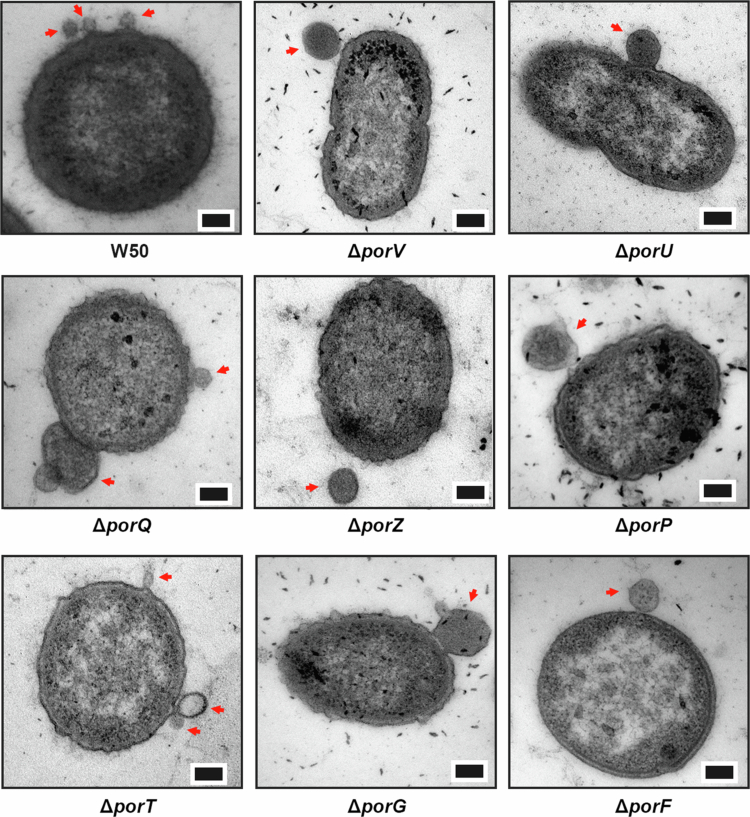
Negative stain TEM of *P. gingivalis* T9SS OMP mutants. Samples were fixed using glutaraldehyde as described in Materials and methods. The scale bar represents 100 nm. OMVs blebbing from cells are highlighted with a red arrow.

**Figure 4.**
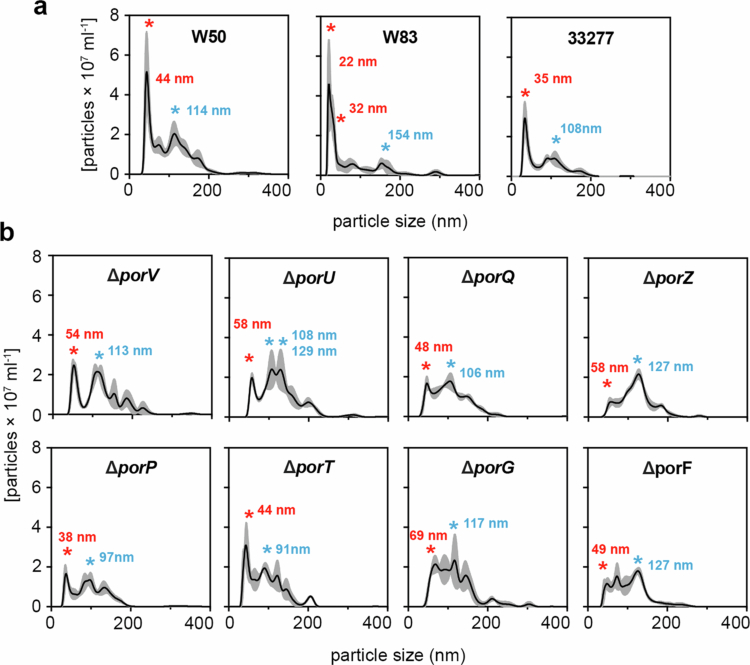
Nanosight analysis of OMVs from *P. gingivalis* W50 and T9SS OMP mutants. NanoSight analysis of crude OMV preparations from (a) W50, W83 and ATCC 33277, and (b) W50 derivative strains. Modes for the smaller and larger OMV species are indicated with a red and blue asterisk, respectively. Data are presented as mean values (black line) +/− SEM (grey area) derived from *n* = 3 biologically independent experiments.

To understand whether these derivative strains also affected the morphology of OMVs as originally observed in the Δ*porV* mutant, we examined them by negative stain transmission electron microscopy (TEM). In line with previous observations [42], a clear electron dense surface layer (EDSL) could be seen on the outer membrane of *P. gingivalis* W50, composed of surface anchored gingipains and capsule, along with OMVs ~30 nm in diameter blebbing from the outer membrane (Figure 3), (**Supplementary Table 4**). On the other hand, while the average cell diameter was consistent with W50, the EDSL was absent in all mutant strains and their OMVs appeared in general much larger with diameters ranging from ~100 to 120 nm. NanoSight single nanoparticle tracking has been used extensively to study vesicle formation in both eukaryotic and prokaryotic systems [61,62] and we used it here to analyse the size distribution of OMV preparations isolated from overnight culture supernatants of W50, but also strains W83 and ATCC 33277 (Figure 4a). In all three strains we observed a clear bimodal distribution of OMV diameters, with an abundant well-defined species <70 nm in diameter, representing smaller OMVs (modes of 44, 22 and 35 nm, respectively), and a species of larger OMVs with a broader range of diameters from ~70 to 200 nm and at lower concentration (modes of 114, 154 and 108 nm, respectively). However, analysis of the W50 mutant strains showed a ~2–5-fold loss of the smaller species but with a similar distribution and concentration of the larger OMVs (Figure 4b).

### T9SS OMP mutants display a loss of lipid A 1-phosphatase activity

**Figure 5.**
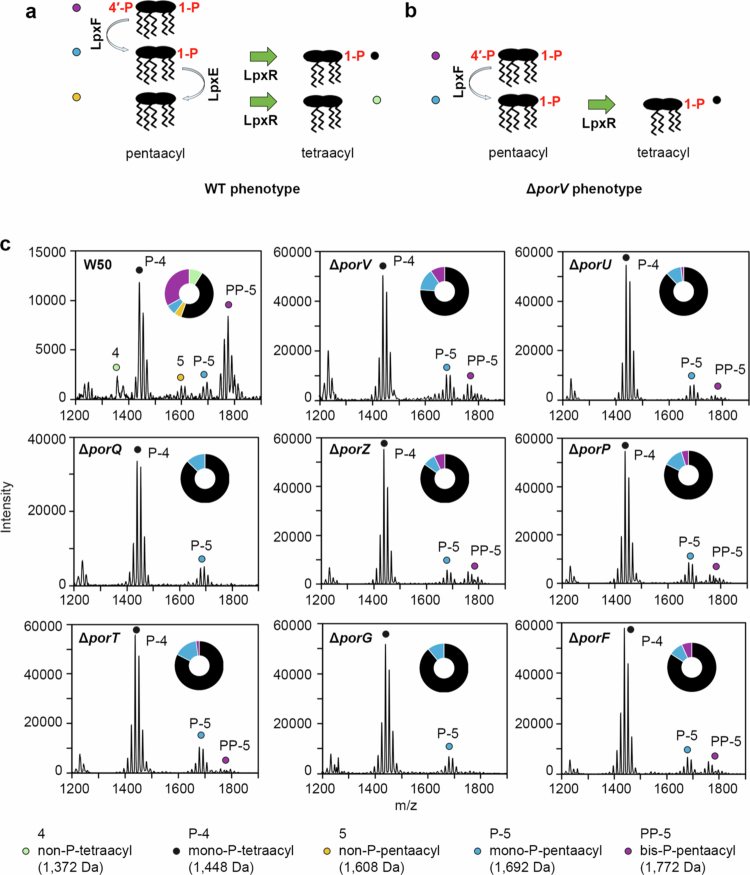
Lipid A modification in *P. gingivalis* T9SS OMP mutants. (a) Schematic showing the lipid A dephosphorylation and deacylation pathway in *P. gingivalis* W50 and (b) Δ*lpxE* mutant. (c) Negative-ion MALDI-TOF MS of lipid A samples highlighting the bis-*P*-pentaacyl, mono-*P*-pentaacyl, mono-*P*-tetraacyl, non-*P*-pentaacyl, and non-*P*-tetraacyl species. Wheel chart shows relative amount of each species.

*P. gingivalis* lipid A is synthesised as a bis-*P*-penta-acyl form but can be subsequently dephosphorylated and deacylated into mono-*P*-penta-acyl, mono-*P*-tetra-acyl, non-*P*-penta-acyl, and non-*P*-tetra-acyl species [42]. This is due to the activities of LpxF1/2 and LpxE, which are expected to be inner membrane enzymes and anticipated to sequentially dephosphorylate bis-*P*-penta-acyl and mono-1-*P*-penta-acyl lipid A species, respectively, with subsequent deacylation in the outer membrane by LpxR to generate tetra-acylated mono-1-*P* and non-*P* lipid A species [39] (Figure 5a). However, in W50 Δ*porV*, non-phosphorylated species are not detected, which indicates that PorV has a role in regulating the activity of LpxE [39] (Figure 5b). Using MALDI-TOF MS we examined lipid A purified from the new mutant strains and compared them to W50. As before, we detected bis-*P*-penta-acyl, mono-*P*-penta-acyl, mono-*P*-tetra-acyl, non-*P*-penta-acyl, and non-*P*-tetra-acyl lipid A species in WT W50, in a ratio of 7:1:9:1:2 while in our Δ*porV* mutant this ratio was 2:3:15:0:0, reflecting a decrease in the bis-*P* species, and an increase in both mono-*P* species, but with no detection of non-*P* species (Figure 5c). Likewise, analysis of the other T9SS mutants showed a similar phenotype to Δ*porV*, although in the Δ*porQ* and Δ*porG* strains there was also no detection of the bis-*P* form. None-the-less, in all mutant strains the mono-*P*-tetra-acyl lipid A species was the predominant form detected.

### LpxE influences vesicle formation and cargo sorting

**Figure 6.**
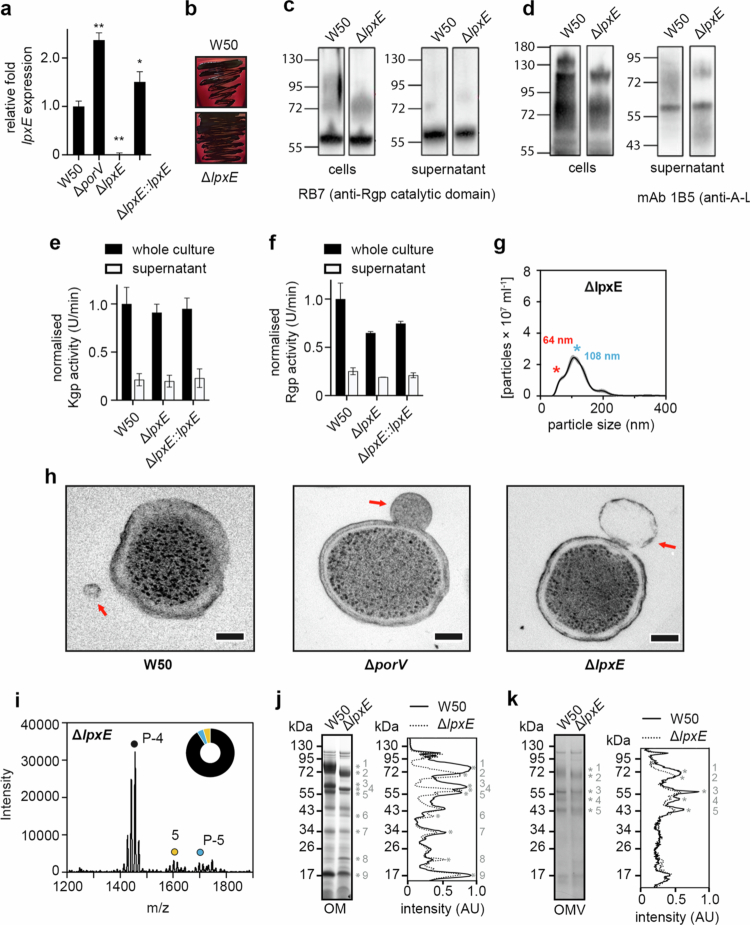
Characterisation of *P. gingivalis* W50 *lpxE* mutant. (a) RT-qPCR analysis of *lpxE* mRNA expression relative to *rgpB* control, in bacteria grown in BHI broth for 6 h. Comparison of WT to Δ*porV*, Δ*lpxE* and Δ*lpxE:lpxE* derivative strains show a strong significant difference by two-tailed Student's *t* test (*p < 0.05, **p < 0.01). Data are presented as mean values +/− SEM derived from *n* = 3 biologically independent experiments. (b) Pigmentation of WT W50 and Δ*lpxE* mutant grown on blood agar plates for 7 days. (c) Western blotting of *P. gingivalis* W50 and *lpxE* mutant cells and supernatants using antisera Rb7 against the RgpA/B catalytic domain or (d) mAb 1B5 against A-LPS, after growth in BHI broth for 24 h. (e) K‐gingipain and (f) R‐gingipain activities in whole cultures and supernatants of *P. gingivalis* W50 and *lpxE* mutant, grown in BHI broth for 24 h. Rgp and Kgp activities were measured using substrates dl‐BR *p* NA and L‐AcK*p* NA, respectively, and are normalised to WT whole cultures. Data are presented as mean values +/− SEM derived from *n* = 2 biologically independent experiments. (g) NanoSight analysis of crude OMV preparation from W50 and Δ*lpxE* strains. Modes for the smaller and larger OMV species are indicated with a red and blue asterisk, respectively. Data are presented as mean values (black line) +/− Standard Error of Mean (SEM; grey area) derived from *n* = 3 biologically independent experiments. (h) Negative stain TEM of W50 WT, Δ*porV* and Δ*lpxE*using high-pressure freezing as described in Materials and Methods. The scale bar represents 100 nm. OMVs blebbing from cells are highlighted with a red arrow. (i) Negative-ion MALDI-TOF MS of lipid A sample isolated from W50 Δ*lpxE* mutant highlighting the major mono-*P*-tetraacyl (*P*-4) species and the minor mono-*P*-pentaacyl (*P*-5) and non-*P*-pentaacyl (5) species. Wheel chart shows relative amount of each species. (j) SDS-PAGE of outer membranes and (k) OMVs isolated from W50 WT and Δ*lpxE* mutant. Densitometry analysis of each lane is shown to the right with changes in peak intensities highlighted with red asterisk and numbered.

We next assessed whether abrogation of lipid A 1-phosphatase activity in the T9SS mutant strains could be due to decreased *lpxE* expression and we evaluated expression of *lpxE* in W50 and Δ*porV* strains using reverse transcription quantitative PCR (RT-qPCR), using *rgpB* for gene normalisation. In addition, we also created a *lpxE* mutant strain in W50 (Δ*lpxE*), and a *lpxE* complementation (Δ*lpxE:lpxE*) and examined these as controls. There was a ~2-fold increase in *lpxE* expression in the Δ*porV* compared with W50, with no expression in the Δ*lpxE* strain and full recovery in the *lpxE* complementation (Figure 6a). This clearly demonstrates that deletion of the *porV* gene does not cause a reduction in *lpxE* expression and loss of lipid A 1-phosphatase activity must be due to other factors.

Further characterisation of Δ*lpxE* showed that unlike the T9SS mutants, this strain has no defect in black colony pigmentation, growth in liquid media, Rgp secretion, A-LPS biogenesis or Lys-gingipain activity (Figure 6b–e, **Supplementary Figure 2**). However, we did observe a ~35% and ~25% reduction in the Arg-gingipain activity in whole cell cultures and isolated culture supernatants, respectively, with activity partially recovered in the Δ*lpxE:lpxE* strain (Figure 6f). Analysis of OMVs isolated from the Δ*lpxE* strain using NanoSight also showed a major loss of the smaller species but not the larger OMVs (Figure 6g). During the previous negative stain TEM analysis of W50 and derivative strains, samples had been fixed using aldehyde cross-linking, which could exacerbate OMV deformation. Therefore, when repeating our TEM analysis with Δ*lpxE*, we prepared samples using high-pressure freezing and used Δ*porV* as a control. As before the average cell diameters were consistent with W50, with blebbing OMVs from W50 and Δ*porV* displaying slightly larger diameters of ~40 and ~120 nm, respectively, however, OMVs blebbing from the Δ*lpxE* strain were much larger with a diameter of ~165 nm (Figure 6h, (**Supplementary Table 5**). Likewise, MALDI-TOF MS analysis of lipid A purified from the Δ*lpxE* strain again showed mono-*P*-tetra-acyl lipid A to be the predominant form observed (Figure 6i).

We finally assessed whether deletion of *lpxE* affected protein accumulation in both the outer membrane and within isolated OMVs. SDS-PAGE analysis of outer membranes and OMVs isolated from W50 and Δ*lpxE* strains showed differences in the band intensities of nine and five proteins, respectively, although these changes were much more pronounced when comparing outer membranes (Figure 6j and k). Of those identified, the top two highest molecular weight bands were of comparable size in both the outer membrane and OMV samples and may indicate the same protein. These differences suggest that the function of LpxE also affects the accumulation and/or modification/molecular mass of specific proteins in both *P. gingivalis* outer membranes and OMVs.

### *P. gingivalis* LpxE represents a new class of lipid A 1-phosphatase

**Figure 7.**
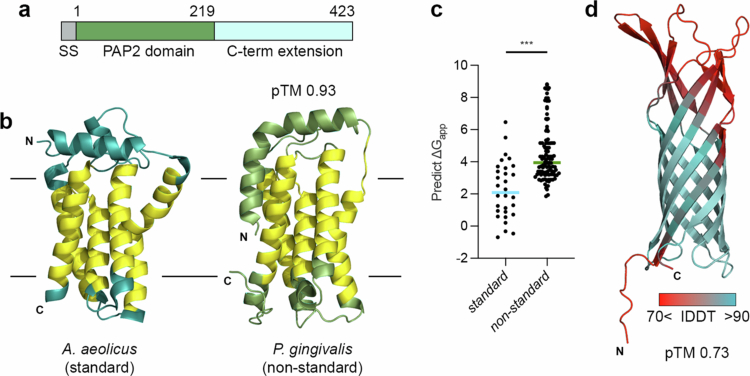
Structural characterisation of *P. gingivalis* like LpxE proteins. (a) Schematic of *P. gingivalis* LpxE highlighting the *N*-terminal signal sequence (SS), *N*-terminal PAP2 domain, and unique extended C-terminal region. Residue numbering is shown above. (b) Crystal structure of *A. aeolicus* LpxE (left; PDB ID: 6ebu) [57] and AlphaFold model of *P. gingivalis* LpxE *N*-terminal PAP2 domain (right; residues 38–219). Transmembrane helices are coloured yellow. (c) Predicted membrane insertion potential (ΔG_app_) of helical transmembrane segments from standard and non-standard (*P. gingivalis* like) LpxE proteins. (d) AlphaFold model of the *P. gingivalis* LpxE C-terminal region (right; residues 220–423).

*P. gingivalis* LpxE is approximately twice the molecular mass of standard LpxE enzymes, and in addition to the standard type 2 phosphatidic acid phosphatase (PAP2) domain it contains a distinct ~200 amino acid extension at its C-terminus [39], formed of *β*-strand secondary structure (Figure 7a, **Supplementary Figure 3**). Using BLAST [50] we identified 21 other bacterial species that contain both a PAP2 domain and an extended C-terminal region, and are >450 residues in total length (**Supplementary Table 3**). While multiple sequence alignment showed high sequence identity in the PAP2 region, this was low in the extended C-terminus (**Supplementary Figure 4**). Further analysis with SignalP [52] revealed a Sec/SPI signal motif at the *N*-terminus of all sequences except that from *Parabacteroides bouchesdurhonensis*. This motif is used in Gram negative bacteria to target proteins to the periplasm or outer membrane and not present in inner membrane proteins. Furthermore, examination of these sequences using DeepLocPro [63], which uses protein language neural network models to detect complex patterns in protein sequences, predicted all *P. gingivalis*-like LpxE proteins to be localised in the outer membrane (confidence range: 58–90%; confidence mean: 75%) (**Supplementary Table 6**).

The potential for a protein sequence to be recognised and inserted into a membrane (ΔG_app_) can be approximated [64] and single spanning integral membrane proteins generally have ΔG_app_ values < 0  kcal/mol [65,66]. However, a recent study suggests that transmembrane helices in outer membrane proteins have higher membrane predicted insertion potentials and this could be a mechanism to prevent membrane insertion during Sec translocation across the inner membrane [67]. We calculated ΔG_app_ values for transmembrane sequences from the PAP2 domain of non-standard *P. gingivalis* LpxE-like sequences and compared them with equivalent regions in other well characterised standard LpxE sequences from *A. aeolicus*, *B. abortus*, *F. novicida*, *H. pylori*, *R. etli* and *C. canimorsus* [57,68–72] (Figure 7b and c, **Supplementary Figure 5**). Although standard LpxE sequences produced a positive predicted ΔG_app_ (mean: ~2 kcal/mol), values for non-standard LpxE sequences were significantly higher (mean: ~4 kcal/mol), implying that these helices have reduced hydrophobicity. Similarly, analysis using DeepTMHMM [54], a deep learning protein language model-based programme used to assign the topology of *α*-helical and *β*-barrels proteins, could not detect many of the transmembrane helices in the PAP2 region of the non-standard LpxE sequences (**Supplementary Table 6**).

We next modelled the *P. gingivalis* LpxE C-terminal extension using AlphaFold [73], which suggested a *β*-barrel structure with highly dynamic surface loops regions (Figure 7d) and this model was consistent across all non-standard LpxE C-terminal regions. While the *P. gingivalis* LpxE PAP2 domain model was predicted with very high confidence (pTM: 0.93), this was lower for the C-terminal region (pTM: 0.73) (Figure 7b and d). However, analysis of non-standard LpxE using DeepTMHMM [54] consistently predicted the presence of eight transmembrane *β*-strands while assessment of protein stability using DeepSoluE [54] indicated that the C-terminal region is not soluble (**Supplementary Table 6**).

## Discussion

Type IX-dependent secretion is a highly complex process and involves at least three stages: (i) cargo recognition in the periplasm and passage through the translocon complex, (ii) cargo release, and (iii) association with the attachment complex and linkage to A-LPS [22]. While the role of PorV, PorU, PorQ and PorZ have been relatively well characterised in relation to the second and third stages, the functions of PorP, PorT, PorG and PorF are unclear [22]. Previous observations in *P. gingivalis* strains W83 and 33277 have demonstrated that PorV, PorU, PorZ, PorT and PorF are essential for gingipain secretion, with deletion of these genes resulting in white colonies forming on blood agar [30,33,74–78]. However, here we have shown that deletion of *porQ*, *porZ* or *porF* in W50 instead results in beige coloured colonies forming on blood agar, although Δ*porV,* Δ*porU*, Δ*porP*, Δ*porT* or Δ*porG* mutants form white colonies, suggesting there may be some variation in the absolute level of decrease in protease secretion/maturation in the former strains.

Our examination of Arg- and Lys-gingipain activities in these mutants also showed a similar phenotype, with no activity detected in the Δ*porV*, Δ*porP*, Δ*porT* and Δ*porG* W50 strains, and high loss of activity in Δ*porQ*, Δ*porZ* and Δ*porF* strains, similar to previous reports for Δ*porT* and Δ*porF* mutants in W83 [78,79]. However, our Δ*porU* mutant displayed full gingipain activity in the supernatant but not in whole cells, suggesting that it is able to export gingipains, but these are not retained on the cell surface consistent with its role as a sortase required for A-LPS attachment. A previously reported Δ*porU* mutant in strain 33277 had no gingipain activity, indicating complete loss of secretion [74]. This is likely due to differences in how the insertion cassettes are designed, resulting in different partial genes being expressed in each of these studies, although both mutants still support the essential role of PorU. We also carried out immunoblot analysis and again our T9SS OMP mutants displayed major secretion defects with an accumulation of pro-gingipains in the cell and supernatant, similar to that described previously for Δ*porV*, Δ*porU* and Δ*porT* mutants in the 33277 strain [74,75].

Disruption of *porV* in W50, *porV* or *porT* in 33277 and *porV*, *porT* or *porZ* in W83 have all been shown to affect A-LPS biogenesis, with an overall reduction in high-molecular weight A-LPS species (A-LPS attached to cargo), and accumulation of lower-molecular weight species in the periplasm [33,75]. Furthermore, higher levels of A-LPS periplasmic retention were observed in the W83 *porZ* mutant compared with the Δ*porV* and Δ*porT* mutant strains, but with no A-LPS detected in the supernatant containing OMVs [33]. Here we show that disruption of *porV*, *porU*, *porP*, *porT*, *porG* and *porF* in W50 in both whole cells and supernatants presents a similar profile with loss of the high-molecular weight A-LPS species. However, disruption of *porQ* caused complete loss of both high and low molecular weight species, while the Δ*porZ* mutant had almost no detectable A-LPS. It is not clear why the Δ*porZ* phenotype is different here, but this could be due to strain differences or again, how the insertion cassettes are designed.

Taken together, these data indicate that these T9SS OMPs have roles during different stages of secretion/attachment. As expected, the Δ*porV*, Δ*porU*, and Δ*porQ/Z* mutants presented distinct phenotypes, reflecting functions related to cargo shuttling and sortase targeting, sortase function, and A-LPS targeting, respectively. On the other hand, the Δ*porP*, Δ*porT* and Δ*porG* mutants have similar phenotypes, and are more likely associated with upstream events. The Δ*porF* mutant was unique and showed no defect in gingipain processing with a similar level of gingipain activity outside of the cell as for the Δ*porQ* and Δ*porZ* mutants. This suggests that PorF may also have a role during the latter stages of secretion, and as a predicted TonB-dependent receptor plug domain-containing protein (UNIPROT ID: Q7MWR1), our data supports a function in importing glycans involved in the A-LPS biogenesis and/or its linkage to secreted cargo [78].

Recent structural and functional studies now indicate that the attachment complex is directly anchored to the PorK/PorN ring on the outer membrane surface [80] and most components of the T9SS are likely contained within one large and dynamic complex. This is also supported by this study, where mutation of eight different T9SS genes are detrimental for type IX secretion, with secretion phenotypes in line with those observed for mutation of the Sov translocon pore, the PorK/PorN ring, and the PorL/PorM proton motive force generator within the translocon complex [5,81]. However, as PorU and PorZ are themselves transported by the T9SS to the bacterial outer membrane surface, the secretion defects reported for Δ*sov*, *ΔporK*, *ΔporN*, *ΔporL* and/or *ΔporM* could be considered to also reflect defects in A-LPS attachment.

We previously demonstrated that a W50 Δ*porV* mutant was unable to produce non-phosphorylated lipid A, [42] and we have now shown that this is also the case for seven further T9SS mutants in W50. Likewise, these mutants also produced larger deformed OMVs when assessed by negative stain TEM, which are comparable to those from a Δ*lpxE* strain and to previously reported Δ*porU* and Δ*porT* mutants in W50 [42,74]. However, hemin-dependent reduction of LpxE activity has also been observed in 33277 strain and it was suggested that this is due to a post-transcriptional or -translational mechanism [39]. Specifically, wild-type 33277 strain grown with 1 μg/ml hemin produced mono-*P*-penta-acyl, mono-*P*-tetra-acyl, non-*P*-penta-acyl, and non-*P*-tetra-acyl lipid A but not the bis-*P*-penta-acyl species, yet with 10 μg/ml hemin only the mono-*P*-tetra-acyl and non-*P*-tetra-acyl lipid A species were detected, which matches the lipid A profile of a Δ*lpxE* derivative grown in 1 μg/ml hemin [39]. In our experiments bacteria were cultured in 5 μg/ml hemin, and we detected all five lipid A species in wild-type W50 and complete loss of all non-*P* species, and a major reduction in the bis-*P*-penta-acyl form of lipid A, in the Δ*lpxE* and T9SS mutant strains, which further demonstrates the influence of exogenous hemin on lipid A 1-phosphatase activity. However, we also observed variation in the relative proportion of the bis-*P*-penta-acyl species in the Δ*lpxE* and T9SS mutant strains, but this can be explained by differences in their growth rates.

Using RT-qPCR we observed no reduction in *lpxE* expression in the *ΔporV* strain which indicates that reduction in lipid A 1-phosphatase activity in the T9SS mutant strains is not due to decreased levels of LpxE. Furthermore, transcriptome analysis comparing *P. gingivalis* W50 grown in the presence of 0.2 and 5 μg/ml hemin has shown that there are no significant changes in *lpx* gene expression under excess hemin conditions, but there is a significant decrease in the expression of several T9SS genes (*porN*, *sov*, *porX*) and predicted type IX cargo protein genes (*inlJ/PG0350*, *PG0410*, *PG0495*, *PG0553*) [82]. PorX is a response regulator protein and part of a two-component system that regulates the expression of T9SS genes [83]. InlJ contains an internalin fold and has been shown to promote mono-species biofilm development while impeding biofilm formation with *Streptococcus gordonii* [84]. PG0401 and PG0553 are both predicted gingipain proteinases [85,86], while PG0495 has a unique sequence with unknown function. Together this strongly implies that a functioning T9SS, rather than PorV alone, is required for LpxE lipid A 1-phosphatase activity in *P. gingivalis*, and this is through the activity of InlJ, PG0410, PG0495, PG0553 or another exported type IX cargo protein.

Using NanoSight, we measured two distinct forms of OMVs in W50, W83 and 33277: a smaller well-defined species and a larger species with a broader range of diameters. In all W50 T9SS mutants we observed a ~2–5 fold reduction in the concentration of the smaller species while the larger species remained relatively constant. Single particle tracking has also been used to monitor OMV size distribution in the *P. gingivalis* strain 381, and has shown that WT 381 again produces both a smaller species and a broader range of vesicle sizes with diameters from ~100 nm, and ~150 to 400 nm, respectively [87]. Moreover, in a Δ*ppaD* mutant strain deficient in peptidylarginine deiminase (PPAD) activity, there was an ~2-fold reduction in concentration of the smaller OMV species but with complete loss of the larger species. PPAD is another cargo of the T9SS, and this could indicate that it has a role in regulating formation of the smaller species in *P. gingivalis*. Although we do not detect loss of the larger species in T9SS or Δ*lpxE* mutant strains this could be due to competing functionality between PPAD and other cargo proteins secreted by the T9SS, differences between strains and/or harvesting OMVs at different growth phases.

Many bacteria express lipid A modifying enzymes, but *P. gingivalis* is unusual as it produces both a lipid A 1-phosphatase (LpxE), two lipid A 4′-phosphatases (LpxF1, LpxF2) and a 3′-*O*-deacylase (LpxR). Furthermore, a triacylated lipid A species has also been observed in W50 OMVs, which is not detected in the Δ*porV* mutant, although no PagL-like 3-*O*-deacylase gene is present in *P. gingivalis* and this likely represents a novel enzyme [42]. Our Δ*lpxE* mutant displayed a different protein profile in the outer membrane and OMVs compared with WT W50, and this suggests that in addition to LpxE being important for OMV blebbing, it also has a role in cargo sorting into OMVs. Moreover, we have identified *P. gingivalis* like LpxE sequences with an extended C-terminal region in 21 additional species with all but one containing a putative Sec/SPI signal sequence. Bioinformatics analyses show conservation in the C-terminus of *P. gingivalis* like LpxE sequences to be low, but this region is also consistently predicted to be insoluble and form a *β*-barrel structure. Although AlphaFold modelling indicated some lack of confidence in the generated models, other analysis strongly predicted that non-standard LpxE proteins are targeted to the outer membrane. *β*-barrel structures are the predominant form of integral membrane proteins that have been detected in the outer membrane of Gram-negative bacteria [88]. However, *E. coli* Wza, involved in capsule transport, is known to form an octameric outer membrane pore through assembly of eight C-terminal transmembrane *α*-helices [89]. Likewise, several components of the type IV secretion system (T4SS) have been identified as containing helical outer membrane segments [90]. There is a significant increase in the predicted membrane insertion potential of outer membrane helices when comparing Wza-like/T4SS to bitopic inner membrane helices [67] and while there is high PAP2 sequence identity across *P. gingivalis* like LpxEs, they also display higher predicted transmembrane ΔG_app_ values when compared to standard LpxE sequences.

**Figure 8.**
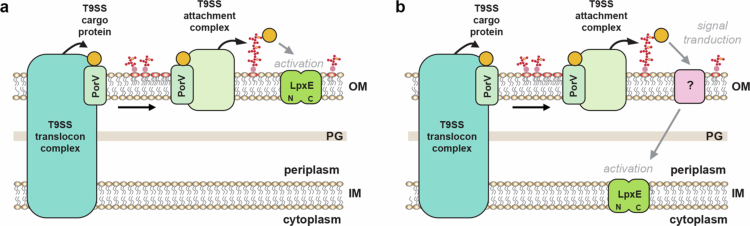
Models for the T9SS dependent activation of LpxE in *P. gingivalis*. (a) A yet to be identified T9SS cargo protein is secreted by the T9SS and attached to A-LPS on the bacterial surface. LpxE is localised in the outer membrane and this cargo protein can directly interact and activate LpxE. (b) LpxE is located in the inner membrane and the surface attached cargo protein instead interacts with with another unknown factor on the bacterial surface, and this results in signal transduction through the periplasm and activation of LpxE located in the inner membrane. Although here the LpxE C-terminal region is drawn within the lipid bilayer its location in relation to the membrane is not known.

Together this suggests that non-standard *P. gingivalis* like LpxE proteins may represent a unique outer membrane localised helical protein with a novel *β*-strand rich C-terminal region, albeit with an unclear structure and unknown function, and experimental studies are now needed to clarify whether these predictions are correct. Nonetheless based on this study we propose two models for the activation of *P. gingivalis* LpxE by the T9SS (Figure 8). In the first model, export of a yet to be identified T9SS cargo protein directly interacts with LpxE located in outer membrane and activates it. In the second model, the secreted cargo protein interacts with another unknown factor on the outer membrane surface, and this results in signal transduction through the periplasm and activation of LpxE in the inner membrane. Of the non-standard LpxE homologues identified, *T. forsythia* and the *Parabacteroides* (*P. faecis*, *P. goldsteinii*, *P. timonensis*) and *Porphyromonas* (*P. gulae*, *P, loveana*) species also contain T9SS component genes and produce OMVs. This indicates that this may be a more general mechanism in other *Bacteroides* that couples type IX cargo sorting with OMV blebbing through the regulation of lipid-A modification.

## Supplementary Material

Supplementary material lpxe_supp_clean
