# Supplementary material for: The Porphyromonas gingivalis lipid A 1-phosphatase LpxE requires a functional type IX secretion system for its activity
Source: J Oral Microbiol. 2025 Dec 14;17(1):2600179. doi: 10.1080/20002297.2025.2600179 (PMC12704123; doi:10.1080/20002297.2025.2600179)
Supplement: Supplementary material — lpxe_supp_clean [file ZJOM_A_2600179_SM2250.docx]

**
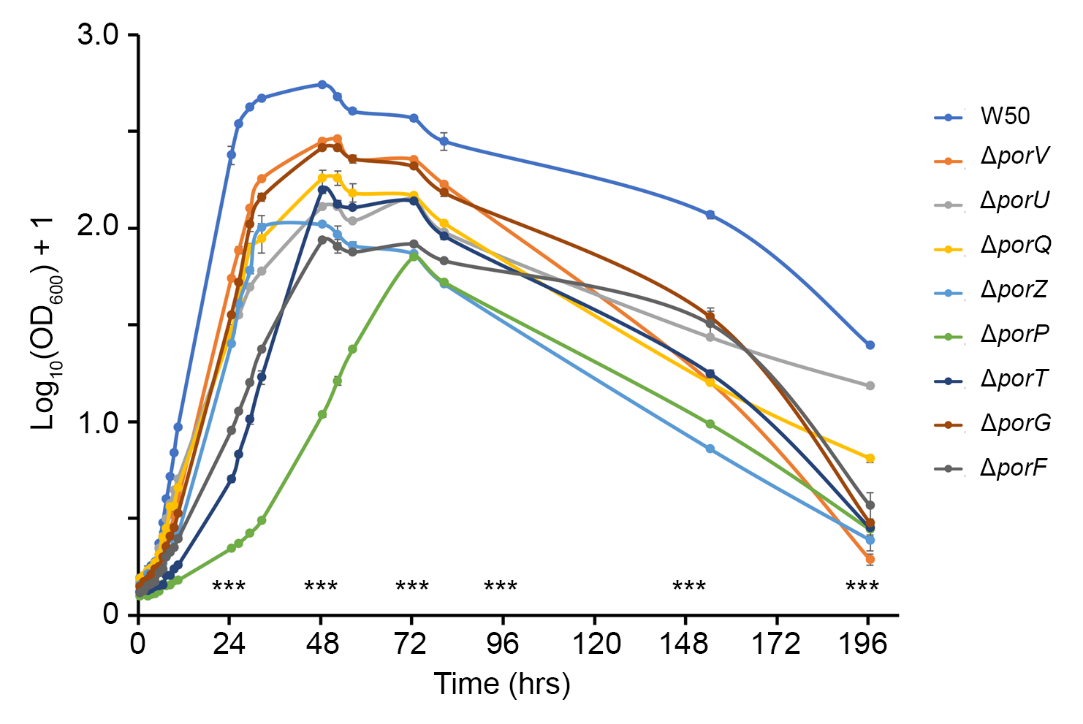
**

**Supplementary Figure 1: Growth curves of *P. gingivalis* W50 and *T9SS* mutants.** W50, Δ*porV*, Δ*porU*, Δ*porQ*, Δ*porZ*, Δ*porP*, Δ*porT*, Δ*porG* and Δ*porF* strains grown in BHI broth supplemented with 5 μg/ml hemin at 37°C. Data are presented as mean values +/- Standard Error of Mean (SEM) derived from *n* = 3 biologically independent experiments. Comparison of W50 WT to Δ*porV*, Δ*porU*, Δ*porQ*, Δ*porZ*, Δ*porP*, Δ*porT*, Δ*porG* and Δ*porF* at 24, 48, 72, 150, and 192 hrs show a strong significant difference by two-tailed Student’s *t* test (*** *p* < 0.001).

**
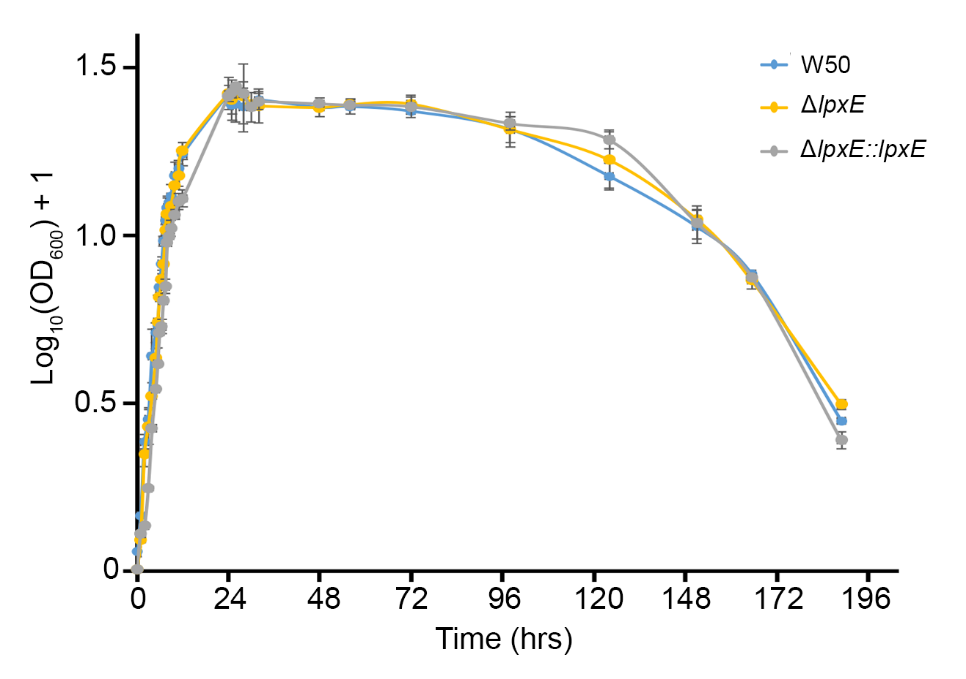
**

**Supplementary Figure 2: Growth curves of *P. gingivalis* W50 and Δ*lpxE* derivatives.** W50, Δ*lpxE* and Δ*lpxE::lpxE* strains grown in BHI broth supplemented with 5 μg/ml hemin at 37°C. Data are presented as mean values +/- Standard Error of Mean (SEM) derived from *n* = 3 biologically independent experiments. Comparison of W50 WT to Δ*lpxE* and Δ*lpxE::lpxE* at 24, 48, 72, 150, and 192 hrs show no significant difference by two-tailed Student’s *t* test (*p* > 0.05).

**
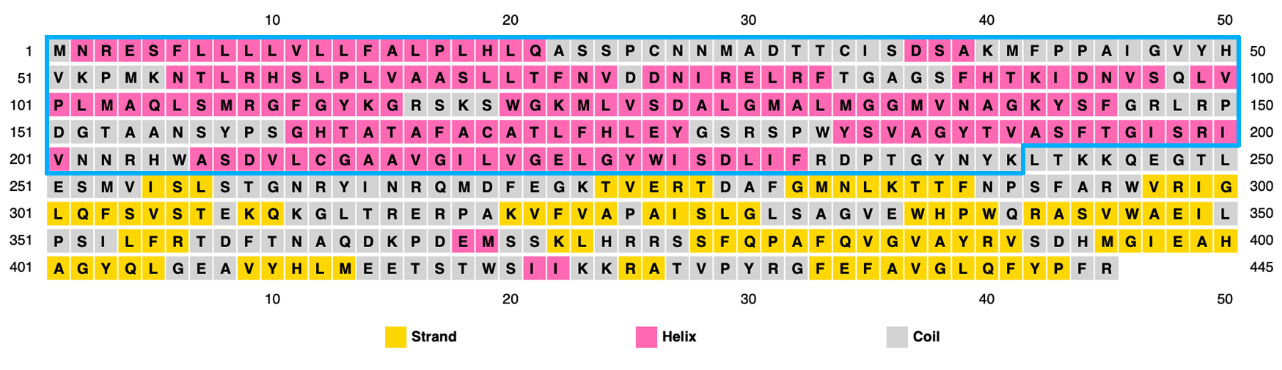
**

**Supplementary Figure 3: Secondary structure analysis of *P. gingivalis* LpxE.** PSIPRED (1) analysis of mature LpxE (no signal peptide) with predicted β-strands coloured yellow, α-helices coloured magenta, and unstructured regions coloured grey. The N-terminal PAP2 region is boxed in blue.

**
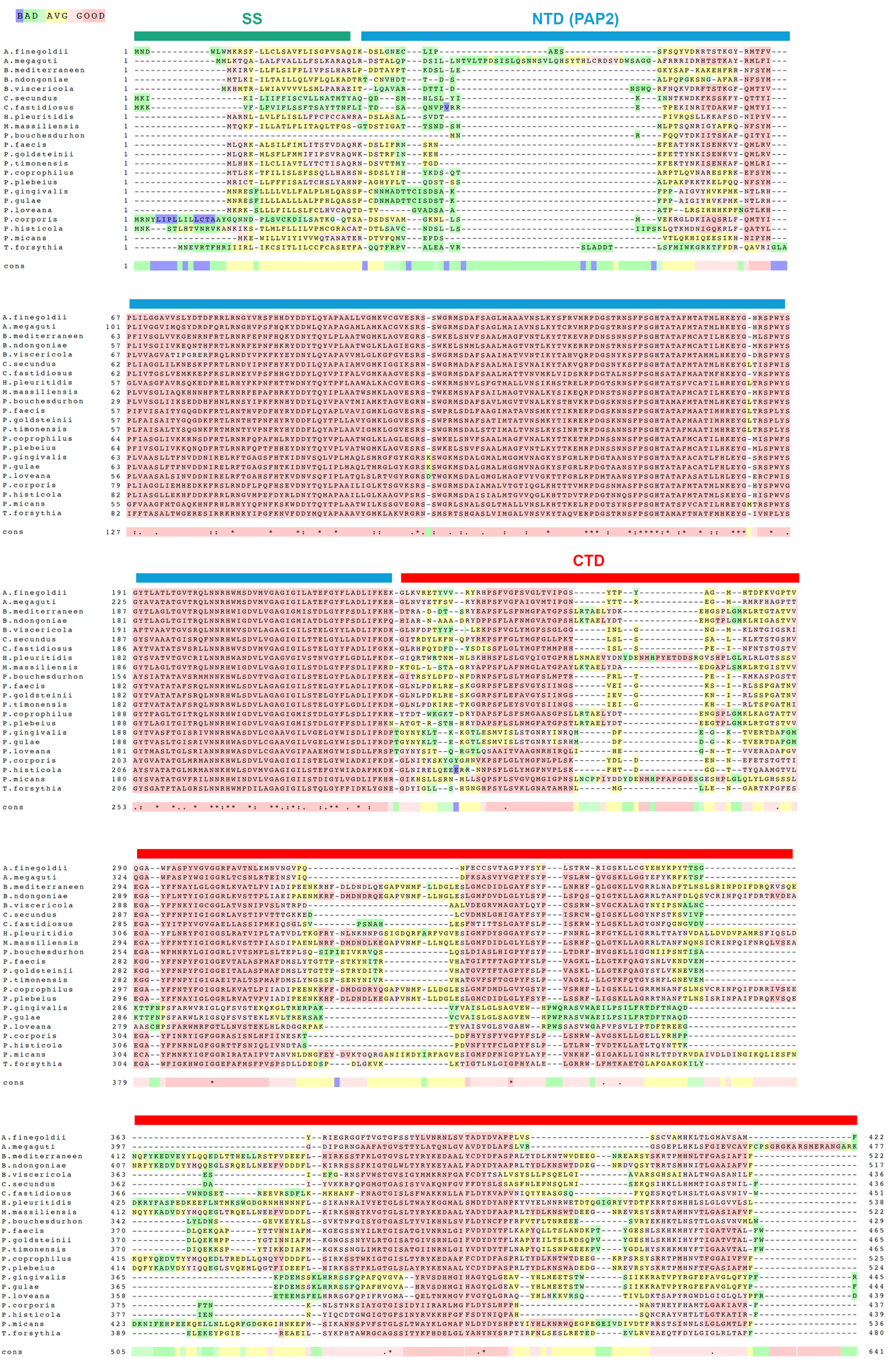
**

**Supplementary Figure 4: Multiple sequence alignment of LpxE homologs with a C-terminal extension.** Reliability of the M-coffee (2) alignment is shown as blue, green, yellow, pink (bad to good). Fully conserved (*) are highlighted, as are residues with strongly (:) and weakly (.) similar properties.

**
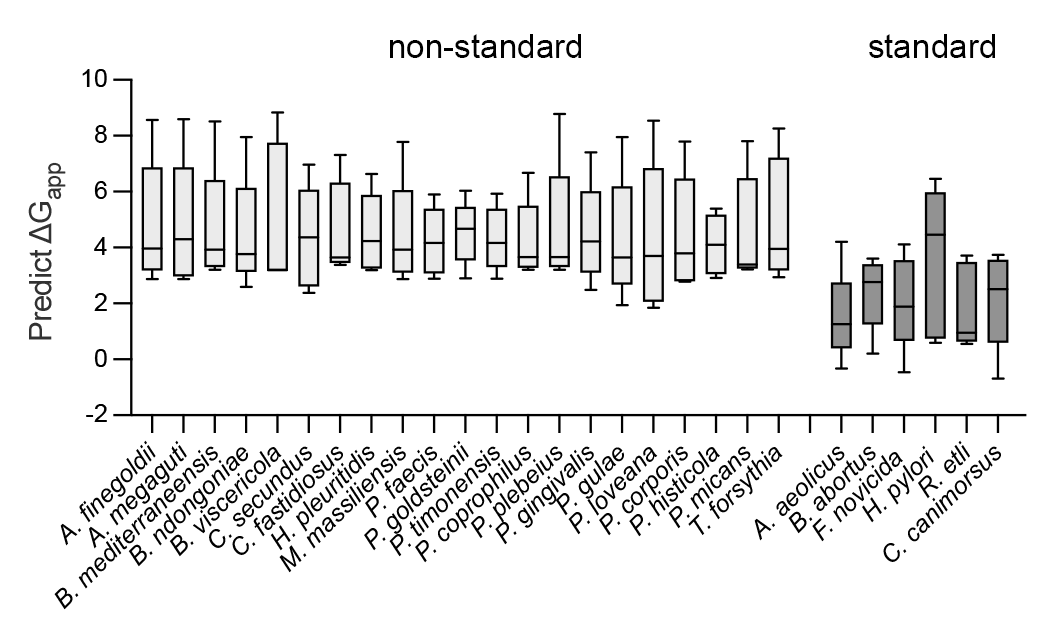
**

**Supplementary Figure 5: Comparison of predicted membrane insertion potentials (ΔG_app_) across LpxE sequences.** The ΔG_app_ was calculated for standard (dark grey) and non-standard (light grey; *P. gingivalis* like) LpxE transmembrane segments. Although some LpxE sequences predict an additional helix at the N-terminus, only the five transmembrane helices that match those in the crystal structure of *A. aeolicus* LpxE (PDB ID: 6ebu) (3) were included for analysis.

**Supplementary Table 1: Strains used in this study**

| **Bacterial strain** | **Serogroup/ genotype** | **Reference** |
| --- | --- | --- |
| *P. gingivalis* W50 | Wild type, no resistance. | (4) |
| *P. gingivalis* Δ*porV* | W50 *porV* (PG0027, PGN_0023) knock-out mutant, Clir. | (5) |
| *P. gingivalis* Δ*porU* | W50 *porU* (PG0026, PGN_0022) knock-out mutant, Clir. | This study |
| *P. gingivalis* Δ*porQ* | W50 *porQ* (PG0602, PGN_0645) knock-out mutant, Clir. | This study |
| *P. gingivalis* Δ*porZ* | W50 *porZ* (PG1604, PGN_0509) knock-out mutant, Clir. | This study |
| *E. coli* BL21 (DE3) | fhuA2 [lon] ompT gal (λ DE3) [dcm] ∆hsdS λ DE3 = λ sBamHIo ∆*EcoR*I-B int::(lacI::PlacUV5::T7 gene1) i21 ∆nin5 | NEB |
| *P. gingivalis* Δ*porP* | W50 *porP* (PG0287, PGN_1677) knock-out mutant, Clir. | This study |
| *P. gingivalis* Δ*porT* | W50 *porT* (PG0751, PGN_0778) knock-out mutant, Clir. | This study |
| *P. gingivalis* Δ*porG* | W50 *porG* (PG0189, PGN_0297) knock-out mutant, Clir. | This study |
| *P. gingivalis* Δ*porF* | W50 *porF* (PG0534, PGN_1437) knock-out mutant, Clir. | This study |
| *P. gingivalis* Δ*lpxE* | W50 *lpxE* (PG1773, PGN_1713) knock-out mutant, Clir. | This study |
| *E. coli* BL21 (DE3) | Competent cells for protein expression, routine T7 expression. | NEB |
|  |  |  |

**Supplementary Table 2: Primer used in this study**

| **Primer** | **Description** | **Sequence (5’ to 3’)** |
| --- | --- | --- |
| porUF1 | Mutagenesis of *porU*. Amplification of *porU* 5′ flanking region (500 bp). | ATGAAACGAATACTTCCAATAGTCGCA |
| porUR1 | of *porU* 5′ flanking region (500 bp). Restriction site SacⅠ is underlined. | ATATATGAGCTCTCATCGTAGTAGTCGATCAAA |
| porUF2 | Mutagenesis of *porU*. Amplification of *porU* 3′ flanking region (500 bp). Restriction site XbaⅠ is underlined. | ATATATTCTAGACCGACCTGACCTACAACCTCA |
| porUR2 | Mutagenesis of porU*.* Amplification of *porU* 3′ flanking region (500 bp). | CTATTGTCCTACCACGATCATTTTCTT |
| porQF1 | Mutagenesis of *porQ*. Amplification of *porQ* 5′ flanking region (300 bp). | ATGATGGAAAAATGTATCTTTGCTCACT |
| porQR1 | Mutagenesis of *porQ*. Amplification of *porQ* 5′ flanking region (300 bp). Restriction site SacⅠ is underlined. | ATATATGAGCTCATGCGAACCACTCATATAAT |
| porQF2 | Mutagenesis of *porQ*. Amplification of *porQ* 3′ flanking region (300 bp). Restriction site XbaⅠ is underlined. | ATATATTCTAGACACTATTTCAAGCGTCTTGTACC |
| porQR2 | Mutagenesis of *porQ*. Amplification of *porQ* 3′ flanking region (300 bp). | TTAGAAGATGCTCTTATCGTCCAA |
| porZF1 | Mutagenesis of *porZ*. Amplification of *porZ* 5′ flanking region (500 bp). | ATGTGCAAAATAAGATTCAGCCTCTTGC |
| porZR1 | Mutagenesis of *porZ*. Amplification of *porZ* 5′ flanking region (500 bp). Restriction site SacⅠ is underlined. | ATATATGAGCTCTTAGTTCCCTTGGCGTAGG |
| porZF2 | Mutagenesis of *porZ*. Amplification of *porZ* 3′ flanking region (500 bp). Restriction site XbaⅠ is underlined. | ATATATTCTAGATCGTCGTGGACAAACTCAATCA |
| porZR2 | Mutagenesis of *porZ*. Amplification of *porZ* 3′ flanking region (500 bp). | TCAGCGAATCACTGCGAAGCG |
| porPF1 | Mutagenesis of *porP*. Amplification of *porP* 5′ flanking region (300 bp). | TTGCATAAATCTTTCCGTTCGCTCA |
| porPR1 | Mutagenesis of *porP*. Amplification of *porP* 5′ flanking region (300 bp). Restriction site SacⅠ is underlined. | ATATATGAGCTCAAAGAGTCCTCTCGTTTCGG |
| porPF2 | Mutagenesis of *porP*. Amplification of *porP* 3′ flanking region (300 bp). Restriction site XbaⅠ is underlined. | ATATATTCTAGAGCATGGCATCCTTCTATCTTTGC |
| porPR2 | Mutagenesis of *porP*. Amplification of *porP* 3′ flanking region (300 bp). | TCAGAGGAAACGAATGCTTTT |
| porTF1 | Mutagenesis of *porT*. Amplification of *porT* 5′ flanking region (300 bp). | ATGCAATTCTTATATAATTCACGTTTTTCG |
| porTR1 | Mutagenesis of *porT*. Amplification of *porT* 5′ flanking region (300 bp). Restriction site SacⅠ is underlined. | ATATATGAGCTCCAGATTGGGCAGAAGG |
| porTF2 | Mutagenesis of *porT*. Amplification of *porT* 3′ flanking region (300 bp). | ATATATTCTAGACGCCTGAACAATATGCG |
| porTR2 | Mutagenesis of *porT*. Amplification of *porT* 3′ flanking region (300 bp). | CTACTCGAAATTGAACGTAAGCAT |
| porGF1 | Mutagenesis of *porG*. Amplification of *porG* 5′ flanking region (300 bp). | ATGAAAACAATTAGTAAGAACCATGCGGC |
| porGR1 | Mutagenesis of *porG*. Amplification of *porG* 5′ flanking region (300 bp). Restriction site SacⅠ is underlined. | ATATATGAGCTCCGCTTGAGCAAAATCGG |
| porGF2 | Mutagenesis of *porG*. Amplification of *porG* 3′ flanking region (300 bp). Restriction site XbaⅠ is underlined. | ATATATTCTAGAGCGGCCGGGTTGTCTTT |
| porGR2 | Mutagenesis of *porG*. Amplification of *porG* 3′ flanking region (300 bp). | CTATTGTTTATTACAAAAAGTCTTACGCAG |
| porFF1 | Mutagenesis of *porF*. Amplification of *porF* 5′ flanking region (500 bp). | ATGAAGGAAGCTATTCCCCGAA |
| porFR1 | Mutagenesis of *porF*. Amplification of *porF* 5′ flanking region (500 bp). Restriction site SacⅠ is underlined. | ATATATGAGCTCTGCGTTACTCCTGCGTA |
| porFF2 | Mutagenesis of *porF*. Amplification of *porF* 3′ flanking region (500 bp). Restriction site XbaⅠ is underlined. | ATATATTCTAGATCAAGCTCTTCGGCGAACT |
| porFR2 | Mutagenesis of *porF*. Amplification of *porF* 3′ flanking region (500 bp). | TTAGAATTCGACGAGGAGACGCA |
| lpxEF1 | Mutagenesis of *lpxE*. Amplification of *lpxE* 5′ flanking region (500 bp). | ATGAATCGAGAAAGCTTTTTACTCCTGC |
| lpxER1 | Mutagenesis of *lpxE*. Amplification of *lpxE* 5′ flanking region (500 bp). Restriction site SacⅠ is underlined. | ATATATGAGCTCAAAGCTGTTGCCGTATGT |
| lpxEF2 | Mutagenesis of *lpxE*. Amplification of *lpxE* 3′ flanking region (500 bp). Restriction site XbaⅠ is underlined. | ATATATTCTAGACTTTCGGCATGAACCTGAA |
| lpxER2 | Mutagenesis of *lpxE*. Amplification of *lpxE* 3′ flanking region (500 bp). | TCAGCGGAAAGGATAGAATTGTAGT |
| lpxEFc | Complementation of *lpxE* | ATATATAGATCTGTATCAGCTATCCGAACAGCAAA GCGAAG |
| lpxERc | Complementation of *lpxE* | ATATATGCGGCCGCTCAGCGGAAAGGATAGAATTGTAGTCCG |
| rtlpxEf | RT-qPCT of *lpxE* | CCTGCCTCTTGTAGCTGCTT |
| rtlpxEr | RT-qPCT of *lpxE* | GCCGAATCCCCTCATCGAAA |
|  |  |  |
|  |  |  |
|  |  |  |

**Supplementary Table 3: LpxE homologs with extended C-terminal regions**

| **Description** | **Species** | **NIH accession code** |
| --- | --- | --- |
| phosphatase PAP2 family protein | *Alistipes finegoldii* | MBD9128102.1 |
| phosphatase PAP2 family protein | *Alistipes megaguti* | WP_232009156.1 |
| phosphatase PAP2 family protein | *Bacteroides mediterraneensis* | WP_083581745.1 |
| phosphatase PAP2 family protein | *Bacteroides ndongoniae* | WP_277122718.1 |
| phosphatase PAP2 family protein | *Barnesiella viscericola* | WP_289539706.1 |
| phosphatase PAP2 family protein | *Coprobacter secundus* | WP_021931000.1 |
| phosphatase PAP2 family protein | *Coprobacter fastidiosus* | WP_302545045.1 |
| phosphatase PAP2 family protein | *Hoylesella pleuritidis* | WP_021583963.1 |
| phosphatase PAP2 family protein | *Mediterranea massiliensis* | WP_289590024.1 |
| phosphatase PAP2 family protein | *Parabacteroides bouchesdurhonensis* | WP_102409487.1 |
| phosphatase PAP2 family protein | *Parabacteroides faecis* | WP_258915275.1 |
| phosphatase PAP2 family protein | *Parabacteroides goldsteinii* | WP_258971860.1 |
| phosphatase PAP2 family protein | *Parabacteroides timonensis* | WP_075559510.1 |
| phosphatase PAP2 family protein | *Phocaeicola coprophilus* | WP_278710372.1 |
| phosphatase PAP2 family protein | *Phocaeicola plebeius* | WP_304122999.1 |
| lipid A C1-phosphatase | *Porphyromonas gingivalis* | WP_005873717.1 |
| lipid A C1-phosphatase | *Porphyromonas gulae* | WP_039437269.1 |
| phosphatase PAP2 family protein | *Porphyromonas loveana* | WP_116678422.1 |
| phosphatase PAP2 family protein | *Prevotella corporis* | WP_277265612.1 |
| phosphatase PAP2 family protein | *Prevotella histicola* | WP_278527811.1 |
| phosphatase PAP2 family protein | *Prevotella micans* | WP_006953444.1 |
| phosphatase PAP2 family protein | *Tannerella forsythia* | WP_080948576.1 |

**Supplementary Table 4: Cell and OMV diameters derived from negative stain TEM of formaldehyde fixed *P. gingivalis* W50 and *T9SS OMP* mutants.** Data presented for WT and derivative strains as mean values ± standard error of mean (SEM) derived from *n* = 9 bacteria or OMVs.

|  | **W50** | **Δ*porV*** | **Δ*porU*** | **Δ*porQ*** | **Δ*porZ*** | **Δ*porP*** | **Δ*porT*** | **Δ*porG*** | **Δ*porF*** |
| --- | --- | --- | --- | --- | --- | --- | --- | --- | --- |
| Mean cell diameter (nm) | 449  ± 30 | 426  ± 33 | 465  ± 18 | 434  ± 27 | 479  ± 30 | 463  ± 34 | 442  ± 32 | 463  ± 25 | 456  ± 35 |
| Mean OMV diameter (nm) | 32  ± 15 | 111  ± 20 | 121  ± 20 | 118  ± 35 | 109  ± 16 | 120  ± 17 | 99  ± 13 | 116  ± 15 | 102  ± 13 |

**Supplementary Table 5: Cell and OMV diameters derived from negative stain TEM of high-pressure frozen *P. gingivalis* W50 and *T9SS OMP* mutants.** Data presented for WT and derivative strains as mean values ± standard error of mean (SEM) derived from *n* = 9 bacteria or OMVs.

|  | **W50** | **Δ*porV*** | **Δ*lpxE*** |
| --- | --- | --- | --- |
| Mean cell diameter (nm) | 434  ± 32 | 448  ± 20 | 460  ± 24 |
| Mean OMV diameter (nm) | 40  ± 23 | 120  ± 12 | 164  ± 14 |

**Supplementary Table 6: Bioinformatic analysis of non-standard *P. gingivalis* like LpxE sequences.** IM = inner membrane, OM = outer membrane.

|  | **DeepLocPro**  membrane localisation probability  (intact sequence) | | **DeepTMHMM**  transmembrane topology prediction  (PAP2 domain) | | **DeepTMHMM**  transmembrane topology prediction  (C-term region) | | DeepSoluEprotein solubility prediction(C-term region) | |
| --- | --- | --- | --- | --- | --- | --- | --- | --- |
|  | IM | OM | helix | strand | helix | strand | Probability^*^ | soluble? |
| *A. finegoldii* | 0.18 | 0.82 | 2 | 0 | 0 | 8 | 0.07 | no |
| *A. megaguti* | 0.21 | 0.78 | 1 | 0 | 0 | 8 | 0.03 | no |
| *B. mediterraneensis* | 0.20 | 0.80 | 4 | 0 | 0 | 8 | 0.41 | no |
| *B. ndongoniae* | 0.22 | 0.77 | 2 | 0 | 0 | 8 | 0.35 | no |
| *B. viscericola* | 0.10 | 0.90 | 2 | 0 | 0 | 8 | 0.03 | no |
| *C. secundus* | 0.34 | 0.66 | 2 | 0 | 0 | 8 | 0.06 | no |
| *C. fastidiosus* | 0.28 | 0.71 | 6 | 0 | 0 | 8 | 0.12 | no |
| *H. pleuritidis* | 0.14 | 0.86 | 2 | 0 | 0 | 8 | 0.15 | no |
| *M. massiliensis* | 0.22 | 0.78 | 4 | 0 | 0 | 8 | 0.46 | yes |
| *P. bouchesdurhonensis* | 0.30 | 0.70 | 5 | 0 | 0 | 8 | 0.07 | no |
| *P. faecis* | 0.18 | 0.82 | 6 | 0 | 0 | 8 | 0.13 | no |
| *P. goldsteinii* | 0.18 | 0.82 | 2 | 0 | 0 | 8 | 0.06 | no |
| *P. timonensis* | 0.19 | 0.81 | 2 | 0 | 0 | 8 | 0.06 | no |
| *P. coprophilus* | 0.21 | 0.79 | 4 | 0 | 0 | 8 | 0.43 | yes |
| *P. plebeius* | 0.19 | 0.80 | 2 | 0 | 0 | 8 | 0.35 | no |
| *P. gingivalis* | 0.39 | 0.60 | 2 | 0 | 0 | 8 | 0.17 | no |
| *P. gulae* | 0.36 | 0.61 | 2 | 0 | 0 | 8 | 0.22 | no |
| *P. loveana* | 0.37 | 0.60 | 1 | 0 | 0 | 8 | 0.10 | no |
| *P. corporis* | 0.18 | 0.82 | 2 | 0 | 0 | 8 | 0.07 | no |
| *P. histicola* | 0.20 | 0.79 | 6 | 0 | 0 | 8 | 0.21 | no |
| *P. micans* | 0.40 | 0.58 | 1 | 0 | 0 | 8 | 0.33 | no |
| *T. forsythia* | 0.23 | 0.74 | 2 | 0 | 0 | 8 | 0.28 | no |

**References**

1. Buchan DWA, Jones DT. The PSIPRED Protein Analysis Workbench: 20 years on. Nucleic Acids Res. 2019;47:W402-W07.

2. Wallace IM, O'Sullivan O, Higgins DG, Notredame C. M-Coffee: combining multiple sequence alignment methods with T-Coffee. Nucleic Acids Res. 2006;34:1692-9.

3. Zhao J, An J, Hwang D, Wu Q, Wang S, Gillespie RA, et al. The Lipid A 1-Phosphatase, LpxE, Functionally Connects Multiple Layers of Bacterial Envelope Biogenesis. mBio. 2019;10.

4. Rangarajan M, Smith SJ, U S, Curtis MA. Biochemical characterization of the arginine-specific proteases of Porphyromonas gingivalis W50 suggests a common precursor. Biochem J. 1997;323 ( Pt 3):701-9.

5. Rangarajan M, Aduse-Opoku J, Hashim A, McPhail G, Luklinska Z, Haurat MF, et al. LptO (PG0027) Is Required for Lipid A 1-Phosphatase Activity in Porphyromonas gingivalis W50. J Bacteriol. 2017;199.
